# Effective Stimulus Parameters for Directed Locomotion in Madagascar Hissing Cockroach Biobot

**DOI:** 10.1371/journal.pone.0134348

**Published:** 2015-08-26

**Authors:** Jonathan C. Erickson, María Herrera, Mauricio Bustamante, Aristide Shingiro, Thomas Bowen

**Affiliations:** Department of Physics-Engineering, Washington and Lee University, Lexington, Virginia, United States of America; University of Tours, FRANCE

## Abstract

Swarms of insects instrumented with wireless electronic backpacks have previously been proposed for potential use in search and rescue operations. Before deploying such biobot swarms, an effective long-term neural-electric stimulus interface must be established, and the locomotion response to various stimuli quantified. To this end, we studied a variety of pulse types (mono- vs. bipolar; voltage- vs. current-controlled) and shapes (amplitude, frequency, duration) to parameters that are most effective for evoking locomotion along a desired path in the Madagascar hissing cockroach (*G. portentosa*) in response to antennal and cercal stimulation. We identified bipolar, 2 V, 50 Hz, 0.5 s voltage controlled pulses as being optimal for evoking forward motion and turns in the expected contraversive direction without habituation in ≈50% of test subjects, a substantial increase over ≈10% success rates previously reported. Larger amplitudes for voltage (1–4 V) and current (50–150 *μ*A) pulses generally evoked larger forward walking (15.6–25.6 cm; 3.9–5.6 cm/s) but smaller concomitant turning responses (149 to 80.0 deg; 62.8 to 41.2 deg/s). Thus, the radius of curvature of the initial turn-then-run locomotor response (≈10–25 cm) could be controlled in a graded manner by varying the stimulus amplitude. These findings could be used to help optimize stimulus protocols for swarms of cockroach biobots navigating unknown terrain.

## Introduction

Insects outfitted with miniature neural-electric stimulation units, so-called biobots, could potentially be utilized as a swarm of mobile agents in a search and rescue operation or other hazardous environment [1]. For example, biobots could be outfitted with a microphone array to map the local environment and to localize a sound source, such as a trapped survivor [2]. Biobotics seeks to take advantage of an insect’s natural centimeter-scale actuator and control mechanisms for navigating uneven and unknown terrain in stable fashion. Additionally, since biobots are typically driven by neural and/or muscular stimulation (e.g., [3, 4]), they can be ≥ 1000-times more power-efficient than state-of-the-art human-made robots [5], which is important for maximizing the amount of time a robotic agent can spend in a remote locale. Various insects have been considered for their aerial and terrestrial biobot potential, including the hawk moth *M. sexta* [6], rhinoceros beetle *M. torquata* [3, 5], American grasshopper *S. americana* [7], and American cockroach *P. americana* [8]. Another attractive candidate for biobot applications is the Madagascar hissing cockroach (MHC) *G. portentosa*. They are excellent climbers, easily reared in the lab, and have a long lifespan (3–24 months). Their relatively large size (length ≈6 cm, mass ≈5 g) is favorable for carrying an electronic backpack without significant disturbance to its natural locomotion. Additionally, the time scale and speed of MHC maneuvers are sufficiently slow such that a human “pilot” or computer-automated system can interact in real-time, sending a sequence of commands to steer it along a desired path.

One straight-forward method for directing locomotion in a MHC biobot is to trigger an escape response (e.g., [9]) via electrical stimulation of the antennae and cerci. Like other cockroach species, the MHC processes tactile (as well as other sensory) antennal inputs to sense and navigate its environment. It is well known that antennal stimulation results in contraversive turns; stimuli to the right antenna causes the insect to turn left, and vice-versa. The cerci, a posterior pair of wind-sensing appendages, are generally used for predator detection. Although MHC cerci are relatively small and wind puffs do not initiate a turn-and-run escape response [10], as is commonly observed with other species (*P. americana*, *B. caniifer*) [9], direct electrical stimulation can still generate a fast forward motion [11].

Prospects for steering the MHC with electric stimuli delivered to both antenna and cerci were initially reported in [11]. A small fraction of test subjects (3 out of 50) exhibited sufficiently strong and sustained responses such that they could be steered along a pre-defined zig-zag path with stimuli delivered through a long (2 m) cable. More recently, a similar experiment has been done with a wireless transceiver “backpack” mounted on the MHC, which allowed for dual neural stimulation and recording capability [12]. The MHC biobot was driven around a pre-defined path in remote controlled fashion, guided either b ad-hoc human command [12] or a Kinect-based automated feedback loop [1, 4]. However, the success rate for steering a MHC biobot around one lap of an S-shaped curve was reported to be relatively low (≈10%) [12]. It has also been recently demonstrated that neural-electric stimuli can be used as a “fenceless” boundary to maintain a MHC within a predefined region to maintain swarm communication [13]. Despite these advances, which stimulus parameters are most effective for evoking a desired and sustained locomotor response in the MHC biobot remain unknown. In addition, a quantitative stimulus-response (S-R) model across a broad stimulus parameter space does not yet exist. Presumably, neural-electric stimulus parameters such as waveform type, amplitude, frequency, and duration can encode surrogate sensory information that is processed by the MHC nervous system to generate a corresponding motor output. Thus, the purpose of this study was to determine effective neural-electric stimulation parameters and a quantitative S-R model that would allow for more robust and precise control of locomotion in *G. portentosa* for biobot applications.

## Material and Methods

### Animals: test subject selection

A colony of MHCs were maintained in a laboratory environment. Infrared heating lamps and daily spraying of tap water helped maintain temperature and humidity to more closely mimic natural habitat conditions. The colony was fed bi-weekly with high-protein food pellets (Carolina Biological). Test subjects were selected from the colony, paying attention to their size and activity levels. We note that no specific permissions were required for use of *G. portentosa* in these experiments as it is a non-endangered, non-protected, invertebrate species. High ethical standards were followed throughout this study.

### Electrode implantation

**Fig 1:**
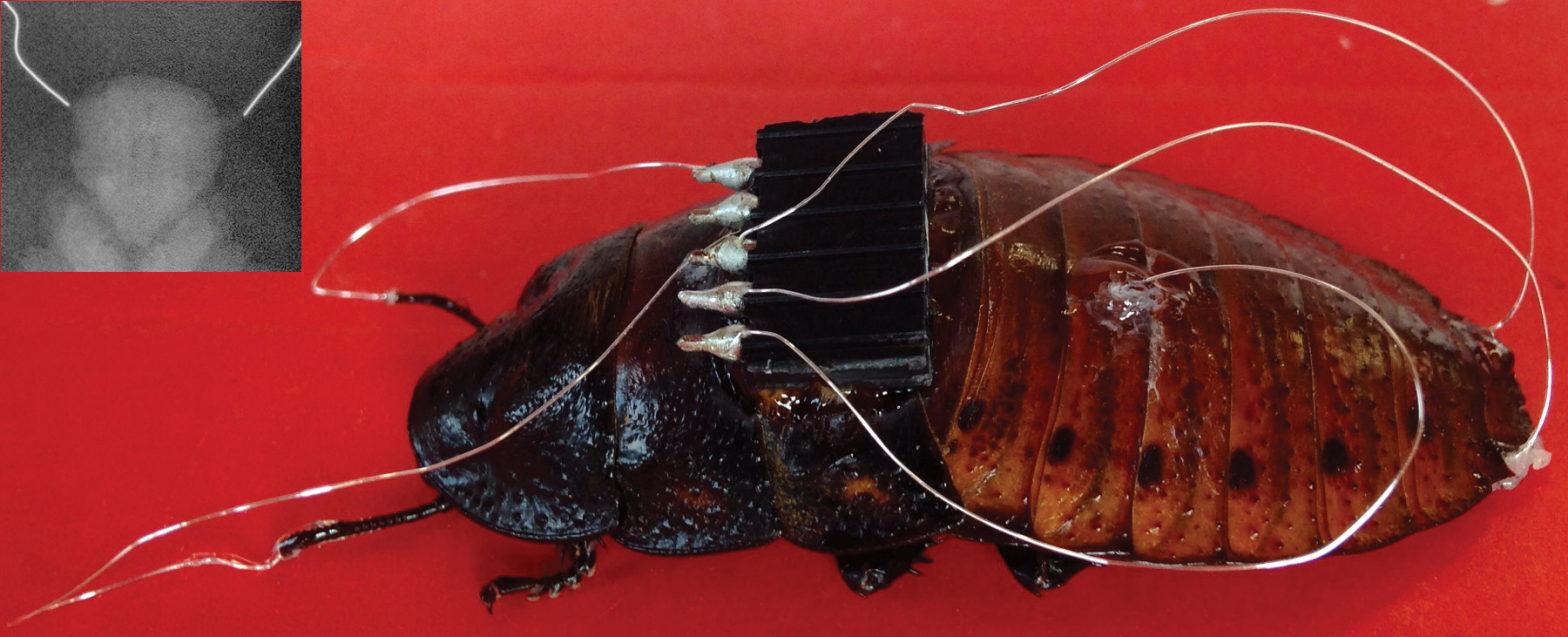
Madagascar hissing cockroach with implanted electrodes. Active electrodes were inserted into antenna and cerci, and the ground electrode into the second abdominal segment. Inset: Ventral view X-ray image highlighting the position of wire electrode tips at the base of antennal sockets. The head is clearly visible in the center of the image.

Electrodes 3–5 cm in length were made from 0.005-inch diameter teflon-coated silver wire (AM Systems, Carlsborg, WA) which was deinsulated on both ends using a flame. One end of each electrode wire was soldered to a 0.1-inch pitch, 5-contact electrical header, and the other end was implanted into the insect (Fig 1). Prior to surgery, insects were anesthetized by cooling with ice packs in a Styrofoam cooler for ≈30 minutes. Antennae were trimmed with a pair of scissors to a length of ≈1 cm. Electrodes were inserted until feeling noticeable resistance, such that the de-insulated electrode tips were positioned near the base of the antennal sockets (Fig 1, inset). Distal tips of both cerci were also trimmed, maintaining as much of them as possible. Electrodes were similarly implanted in cerci, typically about 3–5 mm deep. The ground electrode was inserted at the insect’s midline through the second abdominal segment via a small dorsal puncture made with an insect pin. A drop of super glue (Loctite control gel) was applied to secure each electrode in place, as well as affixing the 5-contact header near the pronotum. The test subject was allowed to recover under a warming lamp for about 30–60 min, until the external temperature, measured with IR thermometer, reached 35 C.

### Stimulus electronics

Custom circuitry to generate current- and voltage-controlled pulses was built based on the design detailed in [14]. Four identical modules were produced, each containing user-selectable current-controlled or voltage-controlled stimulation. The amplitude range of voltage-controlled pulses was 0–5 V, and circuit component values were chosen to set the amplitude range of current controlled-pulses to 0–200 *μ*A. The modules were controlled with two National Instruments (NI; Austin, TX) USB-6251 multifunction devices programmed with LabView. Four independent analog voltage outputs with programmable amplitude, frequency, and duration drove the selected modules. All stimuli were square pulses with 50% duty cycle, either mono- or bipolar. For each stimulus channel, a digital output illuminated a LED during stimulus delivery. Additionally, 8 analog inputs were used to record current flow through, and the voltage across, the active and ground electrodes.

### Motion tracking system

**Fig 2:**
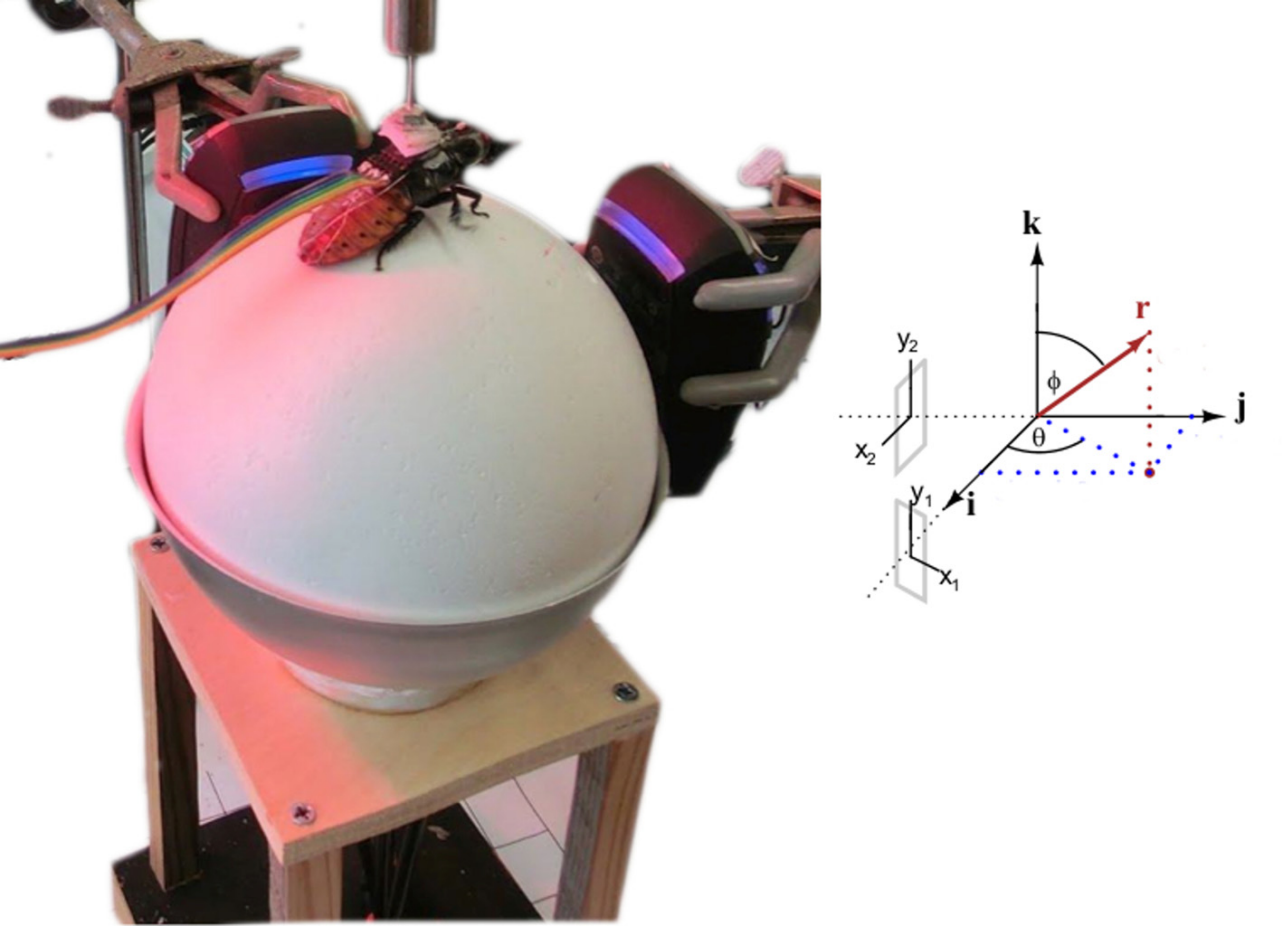
Motion tracking system used to measure stimulus response relationship. Left: Madagascar roach implanted with electrodes sits atop floating polystyrene ball in tethered configuration. Rainbow-colored ribbon cable connects to stimulus electronics. Two orthogonal optical mice measure forward motion and rotation. Image retouched to remove background for clarity. Right: Illustration of optical mice and fictive path coordinate systems. The rotational axis of the trackball *r* is indicated in red.

Test subjects were placed atop a nearly frictionless spherical treadmill and motion was tracked using two optical mice (Fig 2). Similar systems have been described and used by others to track distance and velocity of crickets [15] and American cockroaches [16, 17]. Our spherical treadmill system consists of a 6-inch diameter polystyrene trackball (Smoothfoam, Inc.) floating on a cushion of compressed air. The base was constructed from a 6-inch inner diameter, hemispherical sports ball cake pan (Wilton’s, Inc.) with five air ports for trackball stability (one on-center and four symmetrically off-center). MHCs were mounted with a small patch of velcro attached to the end of a lightweight hollow aluminum rod (1/16-inch outer diameter) that allowed for free rotation (yaw) and vertical translation.

**Fig 3:**
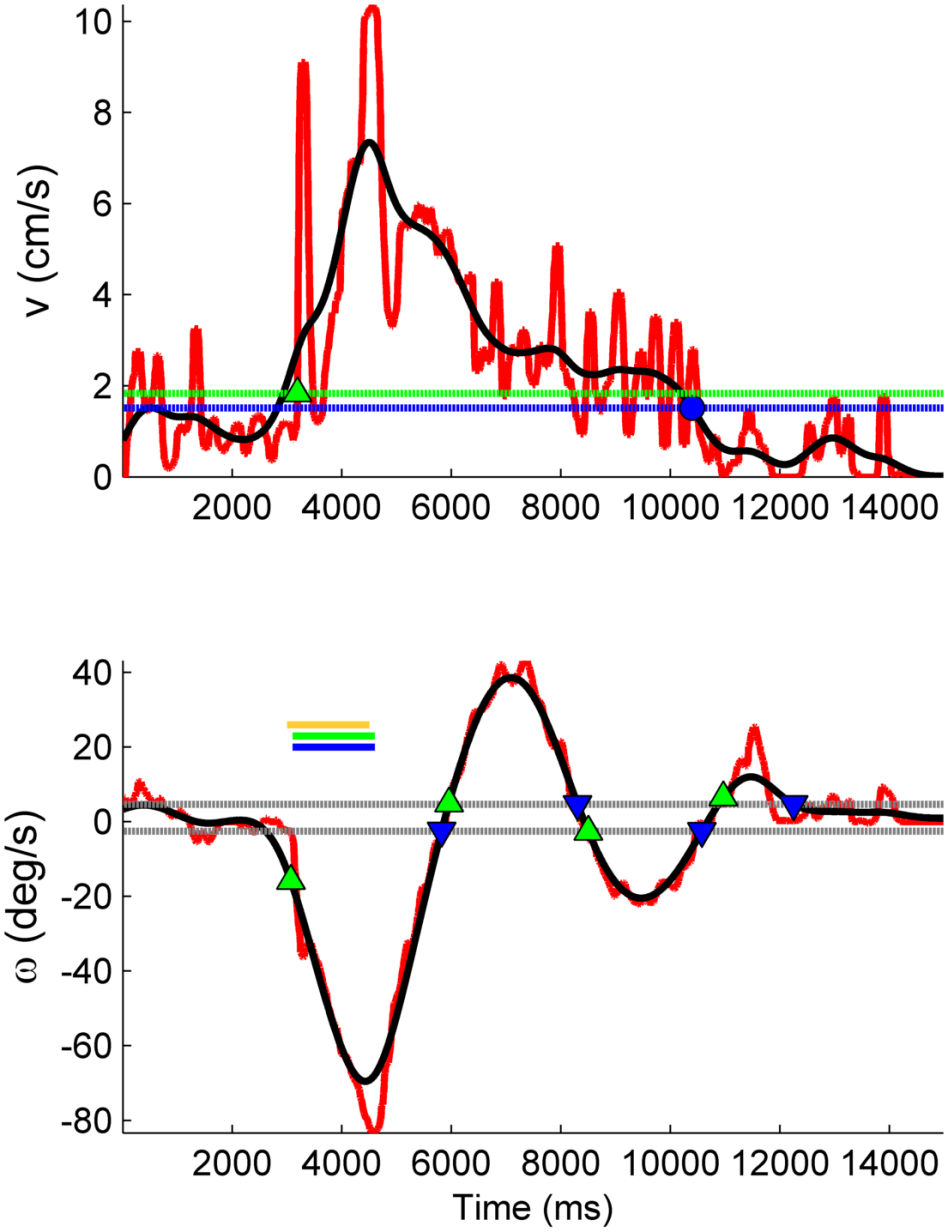
Illustration of automated analysis methods for forward and turning responses. Top panel: Forward walking response. Raw data (red) is low-pass filtered (black) to aid identification of the beginning and termination of the forward response. Dotted green line and diamond mark the response initiation threshold and the beginning of the forward response, *T*_*on*_. Dotted blue line and circle mark the termination threshold and time *T*_*f*_ (see Eq 3). Bottom panel: Turning response. Delivery of stimulus to the left antenna stimulus causes turn to the right (*ω* < 0). Timing of stimuli are indicated by solid horizontal bars (yellow = left antenna; blue and green = right and left cerci). Dotted gray lines: threshold crossings for identifying multiple phases of turning response. Successive pairs of green and blue markers indicate initiation and termination of a single phase of a turning response.

Trackball motion was measured using two USB optical mice (Razer Spectre II, Carlsbad, CA) with high spatial resolution (1800 DPI) and a fast polling rate (500 Hz), aligned orthogonal to each other facing the center point of the trackball, positioned along the equator at a distance of about 2 mm. Custom LabView software (National Instruments, Austin, TX) controlled acquisition and storage of vertical and horizontal displacement in the plane of each mouse’s optical sensor (*x*_*i*_, *y*_*i*_), where *i* indexes the optical mouse (Fig 2). Motion was decomposed into an instantaneous forward walking (or running) velocity *v*(*t*), and turning rate *ω*(*t*), as follows [18]:

$$\begin{array}{c} v\left(t\right)=\sqrt{{\dot{y_{1}}}^{2}+{\dot{y_{2}}}^{2}}\;\;\;\omega \left(t\right)=\frac{\dot{x_{1}}+\dot{x_{2}}}{2R} \end{array}$$ (1)

where *R* is the radius of the trackball, and the dot notation signifies the first time derivative. Discrete time derivatives were computed using a 3-point central difference. In order to facilitate automated analysis, *v*(*t*) and *ω*(*t*) were digitally zero-phase filtered using a 2nd-order Butterworth low pass filter, set for a 3 Hz cutoff (Fig 3).

Motion of the trackball directly corresponds to the virtual fictive path in the Cartesian (***i*** − ***j***) plane, computed as the cumulative sum of the instantaneous heading and path lengths traveled [19]:

$$\begin{array}{c} x\left(t_{k}\right)=\sum_{i=0}^{k}L\left(t_{i}\right)[\cos\theta \left(t_{i}\right)\;i+\sin\theta \left(t_{i}\right)\;j] \end{array}$$ (2)

where ***x***(*t*_*k*_) is the position in the Cartesian plane at time step *k*, *θ*(*t*_*i*_) is the instantaneous heading at time step *i*, and *L*(*t*_*i*_) is the linear distance traveled during the interval *t*_*i*_ to *t*_*i*+1_.

### Experimental paradigms and rationale

Various experiments were designed in this study to determine effective stimulation parameters and to quantify the S-R relationship including:

- Investigating how the locomotor response depends on stimulus amplitude, frequency, and duration
- Comparing the efficacy of voltage-controlled stimuli (VCS) vs. current-controlled stimuli (CCS)
- Comparing the efficacy of charge-balanced bipolar vs. monopolar voltage pulses

Table 1 summarizes these experiments and stimulus waveform parameters used for each. Results from experiments done earlier in the course of this study suggested target parameters used for experiments done later in the sequence.

**Table 1:** Summary of experiments and stimulus parameters. All stimuli were square pulses with 50% duty cycle, with indicated polarity (B = bipolar, positive-first; M = monopolar), amplitude, frequency *f*, and duration *T*_*stim*_. The number of insects tested is indicated by *N*.

Experiment	Polarity	Amplitude	*f* (Hz)	*T*_*stim*_ (s)	*N*
Voltage and frequency dependence	M	1, 2, 3, 4 V	50, 100, 200, 300	1.5	32
Voltage dependence	B	1, 2, 3, 4 V	50,200	1.5	15
Voltage vs. current pulses	B	3, 4 V;50, 100, 150 *μ*A	50, 200	1.5	35
Variable duration	B	3 V	50	0.25, 0.5, 1.0, 1.5	11

Selection of a 1–4 V amplitude range was guided by previous work demonstrating that 3 V was sufficient, in some cases, to evoke repeated motor responses [4, 12]. We tested lower and higher voltage amplitudes to determine whether more power efficient stimuli could generate a similar motor response, and to determine if a higher success rate could be achieved with larger stimuli— i.e., if the threshold stimulus for some tests subjects was > 3 V. Selection of frequencies ranging from 50–300 Hz was motivated by previous work in the *P. americana* species which demonstrated spike rates of ≈50–200 spikes/s in thoracic interneurons in response to tactile stimuli [20]. A stimulus duration of 1.5 s was initially selected based on preliminary tests in our lab, and by a previous study that implemented variable durations of ≈1 s [12]. A 0.25–1.5 s range was tested in a subsequent experiment, guided by durations used by others to remotely steer the MHC around a curved path [4], [12].

We investigated the efficacy of VCS and CCS, as each offers potential advantages. A large majority of previous work on cockroach biobots used VCS (e.g., [4, 8, 13]), which has the practical advantage of requiring simpler electronics hardware compared to CCS. However, the time course of VCS current transients that may drive extracellular neural stimulation (e.g., see Power efficiency) can only be estimated based on the unknown tissue-electrode impedance. CCS offers the theoretical advantage that the time variance of the electric field generated in the insect due to stimulus current can be precisely estimated a priori, and thus more closely matched to the timescale of voltage-gated ion channel kinetics. Current amplitudes of 50, 100, and 150 *μ*A were selected for this study based on previous work using a similar range [11].

Studying the efficacy of bipolar voltage pulses was of particular interest because, to the best of our knowledge, all previous work with cockroach biobots has utilized monopolar stimuli. Since it is has been shown that the cercal and/or antennal system in both *G. portentosa* [11] and *P. americana* [20] habituate to repeated presentations of monopolar stimuli, and that charge-balanced stimuli are known to be less likely to damage electrodes and the surrounding tissue [21], we hypothesized that charge-balanced stimuli could delay or avoid habituation. Additionally, extracellular bipolar VCS have been shown to be about 2.5× more effective than monopolar positive VCS for evoking action potentials in a dissociated cultured neural networks [14].

All experiments were performed by stimulating both cerci and one antenna simultaneously, alternating the left or right antenna in order to avoid any bias. An interstimulus delay between the antenna and cerci of 100 ms was used, based on a similar latency difference of ascending and descending control observed in *P. americana* [16]. For each trial, 3 s of motion data was recorded prior to stimulus delivery to obtain a baseline, and 12 s were recorded post stimulus, sufficient time to observe a clear locomotor response relaxing back to baseline conditions. To limit a single test subject to ≤ 1 hour of locomotion and avoid any potential bias from the insect physically tiring, between 16 and 20 trials were repeated with stimulus parameters values held constant, cycling through all possible combinations listed in Table 1.

### Analysis of forward walking/running

We quantified the S-R relationship by computing the additional distance traveled as a result of the stimulus, Δ*S*, and the change in the average velocity during the response relative to baseline, Δ*v*_*avg*_:

$$\begin{array}{c} \Delta S=\int_{T_{on}}^{T_{f}}v\left(t\right)-{\bar{v}}_{baseline}\;dt\;\;\;\Delta v_{avg}=\frac{\Delta S}{T_{f}-T_{on}} \end{array}$$ (3)

where ${\bar{v}}_{baseline}$ is the mean pre-stimulus velocity, and the integral limits *T*_*on*_ and *T*_*f*_ are the times at which the MHC initiated and terminated a significant forward motion in response to the stimulus (Fig 3, top panel). The threshold “turn on” velocity was defined by $v_{thresh}\geq {\bar{v}}_{baseline}+b_{v}\sigma_{v}$, a user selected constant *b*_*v*_ times the noise level in the pre-stimulus velocity curve, *σ*_*v*_. In practice, we found that *b*_*v*_ = 2 worked well for a variety of velocity curves, and similar results were obtained across a sensible range of values (2 ≤ *b*_*v*_ ≤ 7).

The response termination time *T*_*f*_ was defined as the point where the post-stimulus velocity curve decayed 95% from the peak velocity back toward baseline, or fell below the mean baseline velocity, whichever came first (Fig 3). In rare instances, the walking response did not settle prior to start of the next stimulus delivery, in which case, *T*_*f*_ was set equal to the end of acquisition time of the current trial.

This heuristic method appropriately handled several commonly observed responses: quiescent subject pre-stimulus, with fast rise in velocity following stimulus application followed by a decay back to baseline; spontaneous walker with fast rise in velocity following stimulus application followed by decay to a new, faster or slower walking rate; and spontaneous walker that becomes quiescent following a clear response to stimulus. Note that appropriately marking the end of a response was necessary to accurately compute Δ*v*_*avg*_, but not Δ*S*.

### Analysis of turning

The MHC’s tested often executed a two-phase turning motion in response to a stimulus: an immediate primary turn in a direction contraversive to a stimulus, followed by a secondary “corrective” turn in the opposite direction. Only primary turns were considered for further analysis. For each trial, the primary turning angle and mean change in angular velocity relative to baseline were computed as:

$$\begin{array}{c} \Delta \theta =\int_{T_{1,on}}^{T_{1,off}}\omega \left(t\right)-{\bar{\omega }}_{baseline}\;dt\;\;\;\Delta \omega_{avg}=\frac{\Delta \theta }{T_{1,off}-T_{1,on}} \end{array}$$ (4)

where *T*_1,*on*_ and *T*_1,*off*_ were, respectively, the times at which a primary turn commenced and completed (Fig 3, bottom panel). If no turn was identified during the trial, Δ*θ* and Δ*ω*_*avg*_ were set to zero.

The timing of a turning sequence could be variable even in response to repeated trials with the same stimulus parameters. Therefore, we developed a method to partition the primary and secondary responses that was not sensitive to the exact times at which successive turns were made.

First, significant deviations in *ω*(*t*) relative to a pre-stimulus baseline rate ${\bar{\omega }}_{baseline}$ were identified by marking successive pairs of threshold level crossings, defined as $\omega_{thresh}={\bar{\omega }}_{baseline}\pm b_{\omega }\sigma_{\omega }$ (Fig 3, bottom panel). The noise level *σ*_*ω*_ was computed as the median of the absolute deviation [22], and *b*_*ω*_ was a user-selectable constant. Low values of *b*_*ω*_, in some cases, were more prone to marking false-positives in the turning response, but more accurately estimated the true value of Δ*ω*_*avg*_ and Δ*θ*. In practice, we found that *b*_*ω*_ = 2 worked well for a variety of stereotyped responses.

Second, a slightly modified version of the free split merge expectation maximization (FSMEM) algorithm [14] was applied to cluster primary and secondary turns based on the turning angle and the time at which a particular phase of turn was initiated (S1 Fig). Full details of the modified FSMEM method are available in S1 file.

### Strong responders

A subset of strong responders was selected for further quantitative analysis. Strong responders were defined to be test subjects which exhibited clear suprathreshold, repeatable forward and turning responses to a particular set of stimulation parameters. An additional criterion was that a strong responder must exhibit a primary turn in the expected (contraversive) direction. These criteria were quantitatively identified from the linear and angular velocity response curves as follows:

- Criterion 1: The across-trial median of *v*(*t*) showed a significant transient increase in the post-stimulus epoch, achieving at least the threshold turn on velocity, as defined above (see Analysis of forward walking/running).
- Criterion 2: The extra path length traveled in response to stimulus (defined in Analysis of forward walking/running) was at least one test subject body length: Δ*S* > *L*_*roach*_.

**Fig 4:**
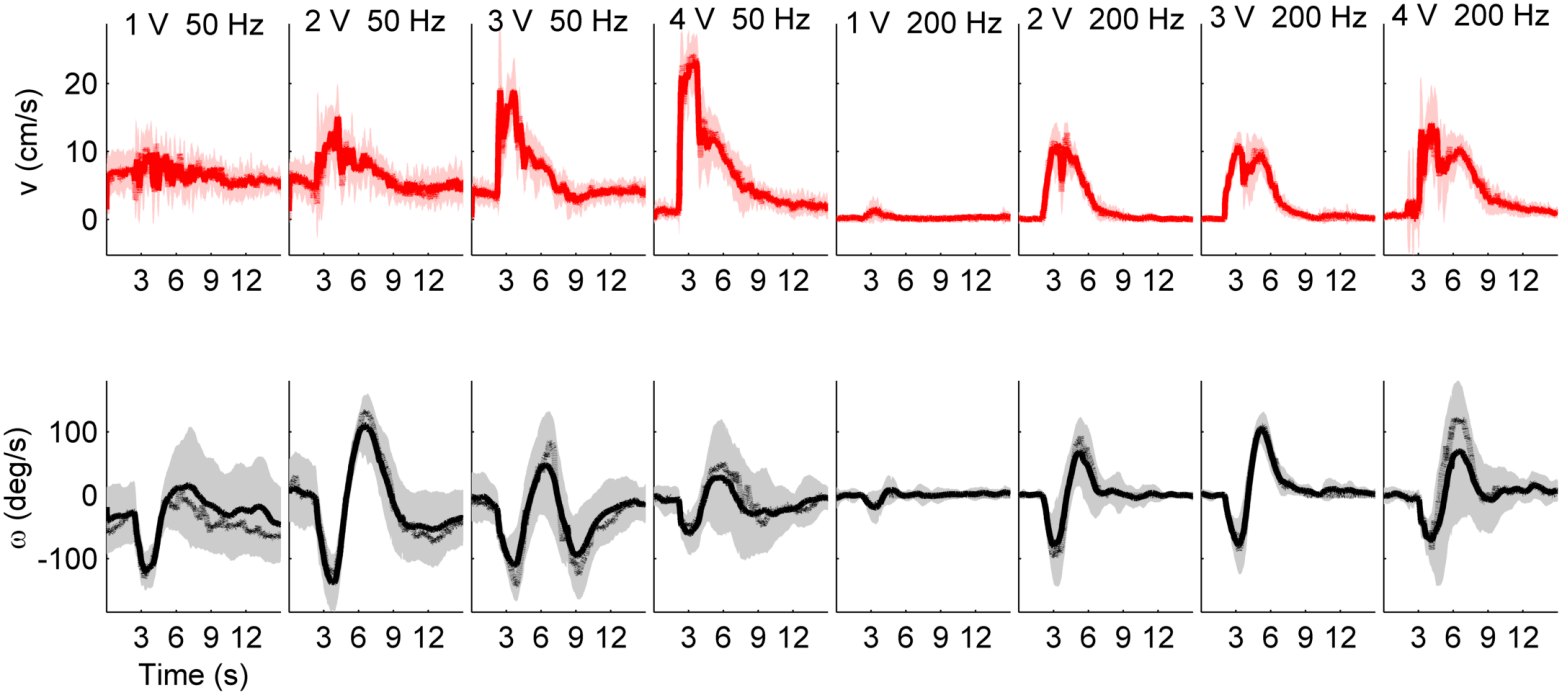
Example of trial-averaged responses of linear and angular velocity vs. time for stimuli delivered to both cerci and left antenna. Turns to the right are indicated by *ω* < 0; turns to the left by *ω* > 0. Each column corresponds to a particular stimulus parameter combination. Solid and dotted curves: mean and median across all trials, respectively. Shaded area marks one standard deviation of trial-averaged responses. Each trial lasted for 15 s, and the the stimulus duration was 1.5 s. The subject was spontaneously walking during 1–3 V, 50 Hz trials, but not others. Repeated strong forward walking responses with turns in the proper direction were observed for all stimulus parameter combinations, except for 1 V, 200 Hz.

For example, Fig 4 illustrates a test subject identified as a strong responder to all combinations of voltage and frequency tested, except for the fifth (1V, 200 Hz). The response to the fifth stimulus combination was essentially a brief, insignificant forward jerk. Such a response passed strong responder criterion 1 due to the pre-stimulus velocity profile being nearly flat and noiseless, but failed on the criterion 2. By contrast, all other stimuli evoked a clearly more prolonged walking motion during which the MHC traveled a significant distance forward.

## Results

### Thresholds for amplitude and duration

**Fig 5:**
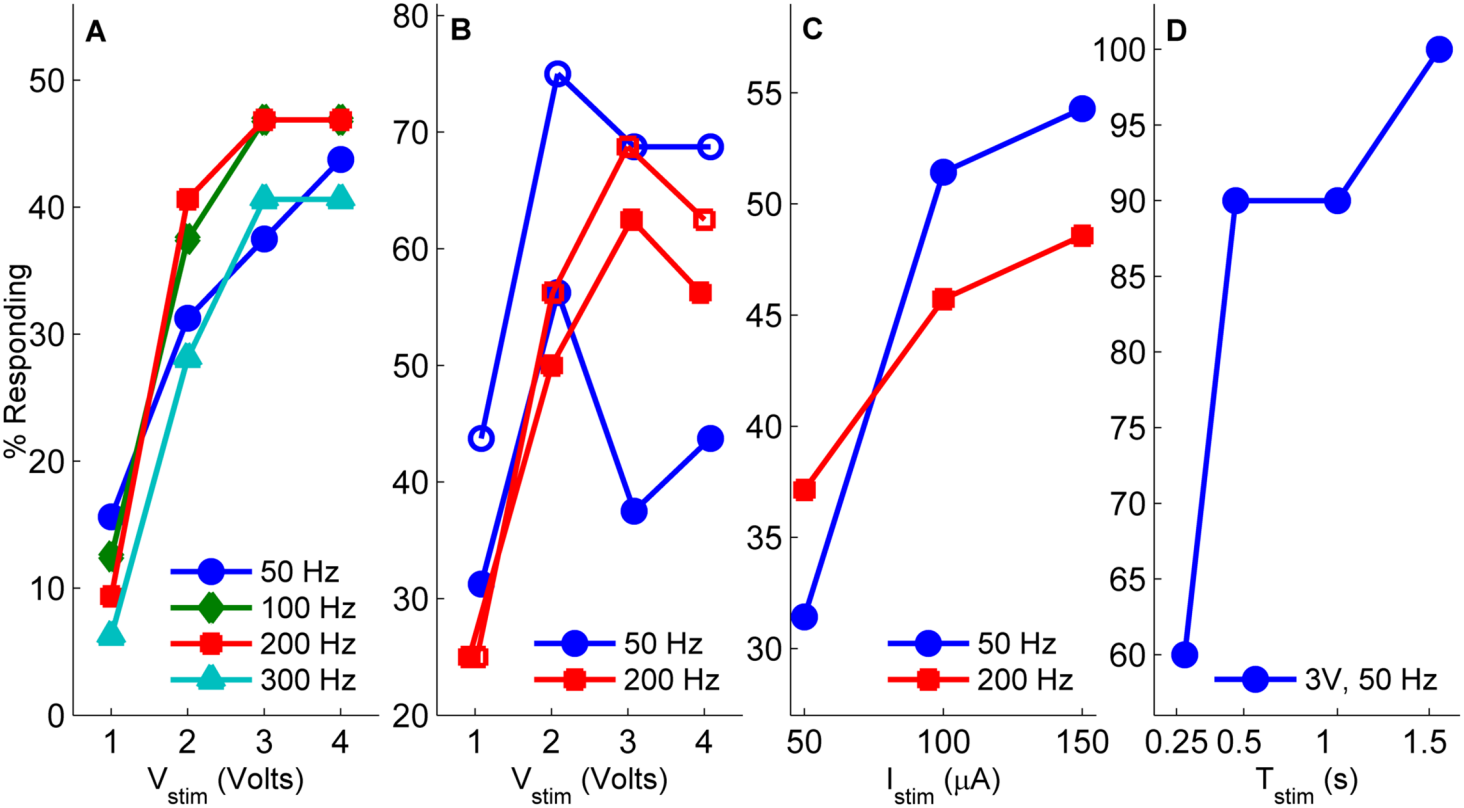
The percent of test subjects responding to different stimulus types: Voltage controlled monopolar positive voltage pulses (A); charge-balanced voltage (B) and current controlled pulses (C); different durations with set voltage and frequency (D). In (B) open marks indicate only that a clear forward response was observed, whereas closed markers additionally indicate turning in the proper (contraversive) direction.

Results to identify optimal stimulus amplitude, frequency, and duration are illustrated in Fig 5. The percent of strongly responding test subjects are shown for a particular stimulus parameter combination. All results reported are for a 1.5 s duration (panels A-C), except for when this parameter was varied (panel D).

For monopolar positive voltage pulses (Fig 5A), maximal response rates of 41–47% were achieved. Larger amplitudes were more effective at eliciting motion from a greater number of test subjects. In most cases, the maximal response rate was achieved starting at 3 V, with no further gains realized by increasing the stimulus amplitude. No strong frequency dependence was observed, however 100 and 200 Hz stimuli were slightly more effective.

For bipolar voltage pulses, the maximal number of strong responders (Fig 5B, solid marks) was achieved at amplitudes of 2 V for 50 Hz (56%), and 3 V for 200 Hz (63%). For 50 Hz stimuli, while ≈70% of test subjects responded with significant forward motion to the highest amplitudes tested (Fig 5B, open marks), about half exhibited turns in the wrong direction, accounting for the dip noted at 3 and 4 V (Fig 5B, closed marks). For 200 Hz stimuli, the decrease between 3 and 4 V resulted from some test subjects exhibiting a bucking spasm while splaying their legs, instead of coordinated motion.

For bipolar current pulses, the fraction of strong responders was positively correlated to amplitude (Fig 5C), with a maximum success rate of 54% achieved with 150 *μ*A, 50 Hz stimuli. Similar to voltage pulses, no strong frequency dependence was noted, although 50 Hz pulses were slightly more effective than 200 Hz. It is worth noting that an asymptote in the percent of stronger responders does not appear to be reached within the magnitude range tested; higher current amplitudes could possibly achieve higher success rates.

For variable duration with bipolar VCS (Fig 5D), a marked increase in the number of strong responders was observed between 0.25 and 0.5 s, with an additional small increase observed for 1.5 s. Only test subjects that were previously observed to be strong responders to 1.5 s stimuli were included in this experiment, hence the maximal 100% rate of strong responders reported in Fig 5D). A possible neurobiological basis for this result is provided in the Discussion section.

### Habituation: monopolar vs. bipolar pulses

**Fig 6:**
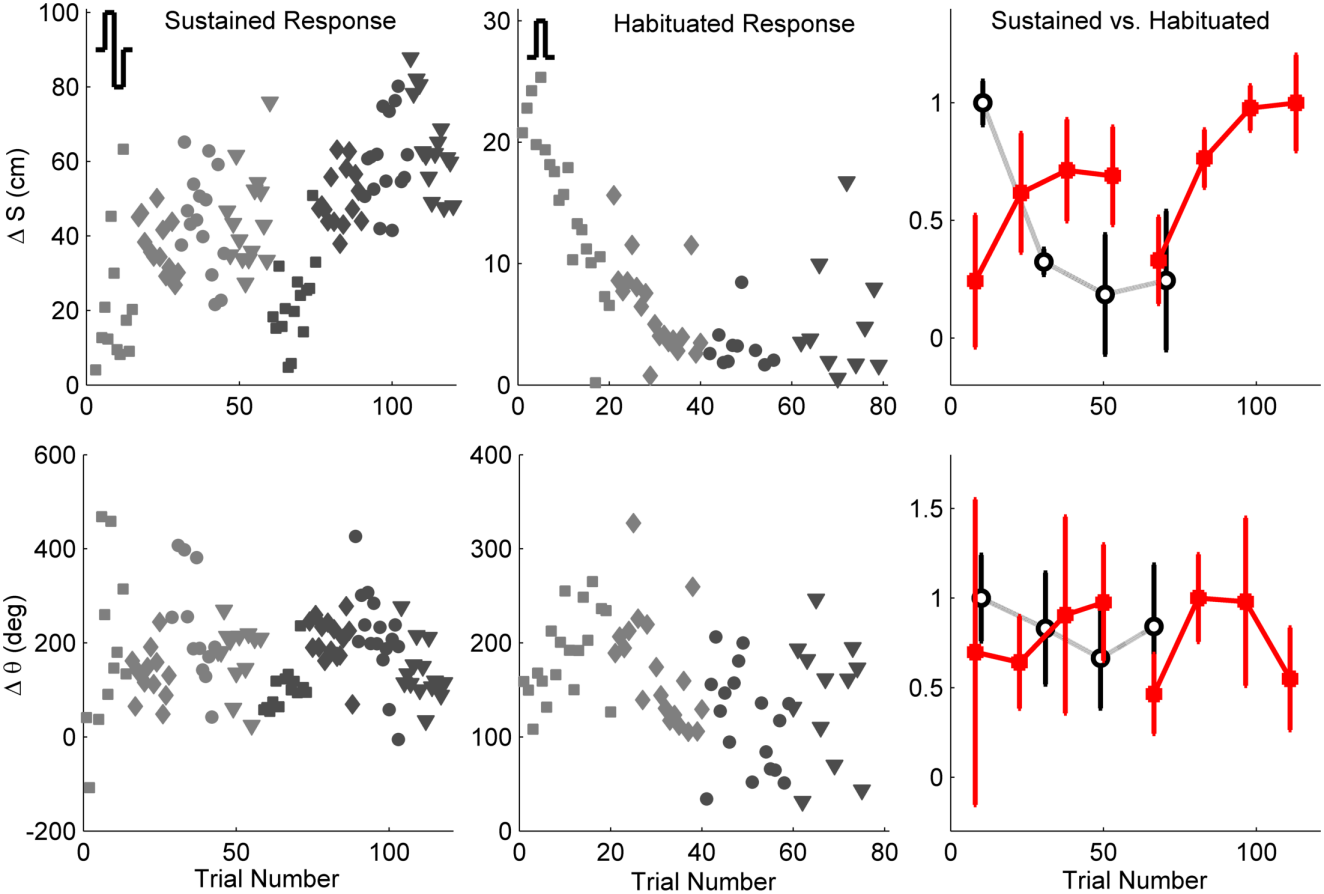
Comparison of sustained response with bipolar stimuli (left column) and habituated response (center column) with monopolar stimuli. Additional path length (top row) and turning angle (bottom row) in response to successive trials are shown. Marker coding:{□, ⋄, ∘, ▿} = {1,2,3,4} V and {light gray, dark gray} = {50, 200} Hz. For ease of comparison, normalized responses (median ± S.D. computed for each stimulus type) vs. trial number for sustained (red, filled marks) and habituated (black, open marks) are shown in the right column.

An example comparison of sustained and habituated locomotion responses to bipolar and monopolar stimuli, respectively, is illustrated in Fig 6. In this case, bipolar stimuli (left column) evoked relatively stable responses both for forward (Δ*S*) and turning (Δ*θ*) motion over 120 trials, so long as the amplitude was above a threshold value (≥ 2V). By contrast, for monopolar stimuli, Δ*S* decreased ≈80% after 40 successive trials (Fig 6, middle column). The turning response Δ*θ* also decreased, but to a lesser degree (≈30%). However, a simple turn by itself without any significant forward motion would not be useful in practice. In general, a habituating locomotor response was characterized by a gradual or sudden decrease in forward locomotor response to repeated presentations of stimuli, often occurring after 20–80 stimuli.

The outcomes of monopolar vs. bipolar stimuli are summarized in Table 2. To quantitatively compare their overall efficacy, a ratio *η* was computed as follows:

$$\begin{array}{c} \eta =\frac{N_{sr}}{N_{hab}}. \end{array}$$ (5)

In Eq (5), *N*_*sr*_ is the maximum number of strong responders averaged across all frequencies tested *N*_*sr*_ (see Fig 5), and *N*_*hab*_ is the number of habituated test subjects. A higher value for *η* indicates a more effective stimulus type. By this measure, bipolar stimuli were nearly 14× more effective, primarily because they were 11× less likely to lead to a habituated response.

**Table 2:** Comparison of monopolar vs. bipolar voltage stimuli strong responders and habituated test subjects. *N* = number of test subjects; *N*_*hab*_ = percent of subjects that exhibited habituated response; *N*_*sr*_ = mean ± S.D. of maximal number of strong responders; and *η* = *N*_*sr*_/*N*_*hab*_. Overall, bipolar stimuli were found to be much more effective.

Stimulus Type	*N*	*N*_*hab*_ (%)	*N*_*sr*_(%)	*η*
Monopolar	32	31.3	46.9 ± 3.0	1.5 ± 0.1
Bipolar	35	2.9	59.4 ± 4.4	20.5 ± 1.5

### Dependence of locomotor response on amplitude, frequency, and duration

**Fig 7:**
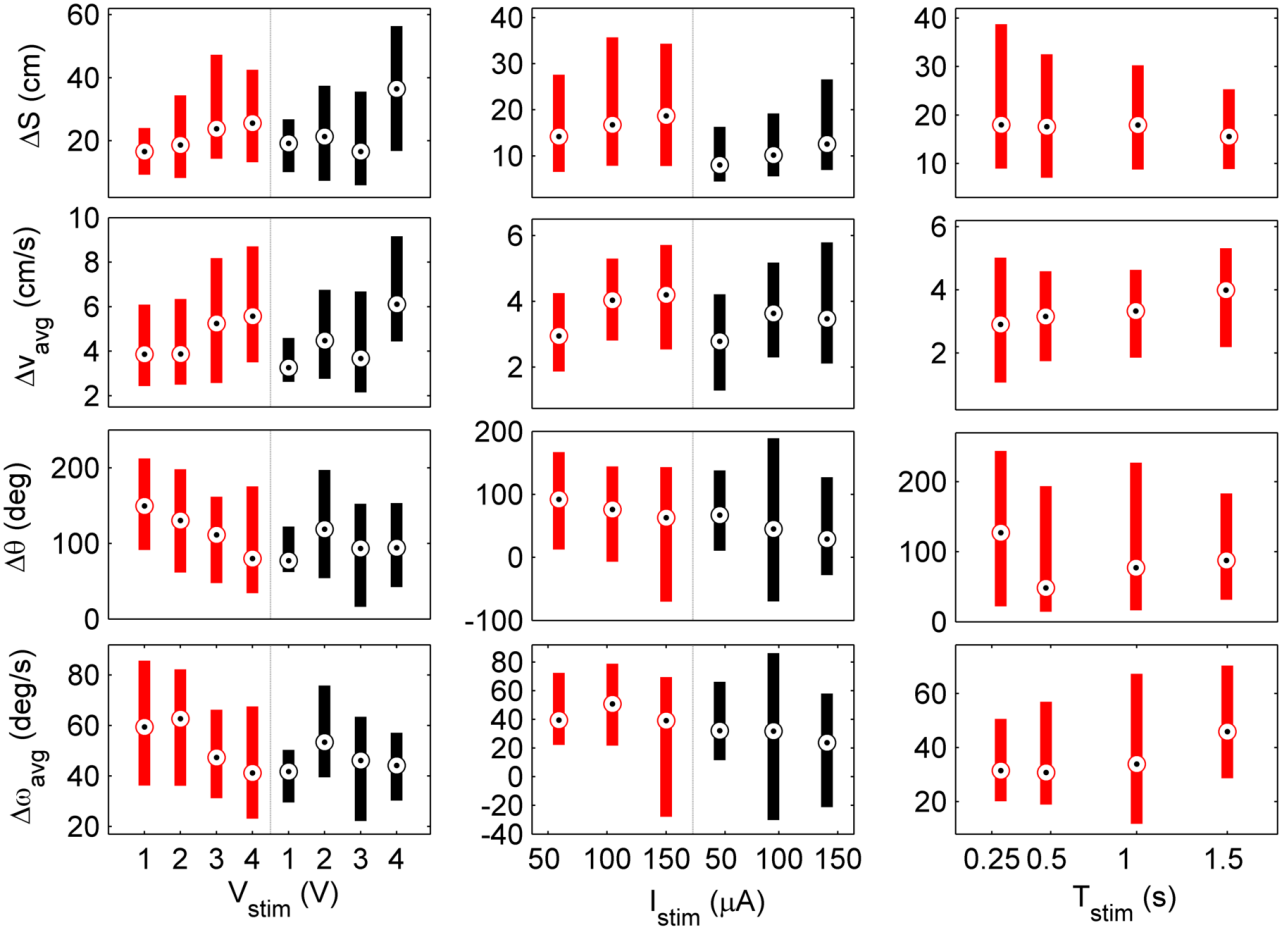
Locomotion response as a function of VCS (left) and CCS (center) bipolar stimulus amplitude, and stimulus duration (right). Red and black color-code stimulus frequencies of 50 Hz and 200 Hz, respectively. Bulls eye markers indicate the median, and bars cover the 25–75th percentile. Variable amplitude and duration tests had constant *T*_*stim*_ = 1.5 s, while variable duration tests were done with constant 3V, 50 Hz.

Fig 7 shows how locomotion depended on the amplitude of bipolar VCS and CCS, as well as stimulus duration. Results were collated across the subset of test subjects that were identified as strong responders to a particular stimulus parameter combination. The statistical dependence of the locomotor response on stimulus amplitude or duration are summarized in Table 3. Statistical significance was assessed by computing the Pearson correlation coefficient *r* (range of -1 to 1) and associated *p*-value (the probability of getting a correlation as large as the observed value *r* by random chance, when the true correlation is zero; *H*_*o*_: no correlation) [23]. Additionally, the linear regression slope *m* was computed whenever *p* < 0.06 (value chosen based on gap between small and large *p*-values given in Table 3).

**Table 3:** Statistical tests for correlation of locomotion response metrics to stimulus amplitude or duration. Variables in each column are: number of responses analyzed to 50 Hz and 200 Hz stimuli, respectively *n*_50_ and *n*_200_; stimulus frequency *f*; Pearson correlation coefficient *r* and associated *p*-value; and linear regression slope *m*, computed only when a significant correlation was identified (*p* < 0.06). Units of *m* are given in the physical units of the metric (e.g., cm) per 1 V for voltage pulses, per 50 *μ*A for current pulses, per 1s for variable duration tests.

		Voltage Pulses			Current Pulses			Var. Duration		
		*n*_50_ = 460			*n*_50_ = 591			*n*_50_ = 456		
		*n*_200_ = 489			*n*_200_ = 551					
Metric	*f* (Hz)	*r*	*p*	*m*	*r*	*p*	*m*	*r*	*p*	*m*
Δ*S*	50	0.23	1e-8	4.66	0.21	6e-9	5.29	-0.07	0.14	–
(cm)	200	0.36	2e-20	6.90	0.21	7e-9	3.9			
Δ*v*_*avg*_	50	0.24	3e-10	0.75	0.27	8e-14	0.85	0.16	7e-5	0.93
(cm/s)	200	0.43	3e-31	1.30	0.26	9-e13	0.82			
Δ*θ*	50	-0.24	2e-7	-21.2	-0.11	7e-3	-20.1	0.05	0.29	–
(deg)	200	-0.03	0.48	–	-0.09	4e-2	-15.0			
Δ*ω*_*avg*_	50	-0.18	1e-4	-7.70	-0.21	4e-7	-15.3	0.13	7e-3	13.8
(deg/s)	200	0.03	0.48	–	-0.18	1e-5	-14.0			
*R*_*c*_	50	0.15	0.37	–	0.28	6e-2	5.9	-0.04	0.80	–
(cm)	200	0.40	2e-2	0.47	0.27	6e-2	25.1			

**Fig 8:**
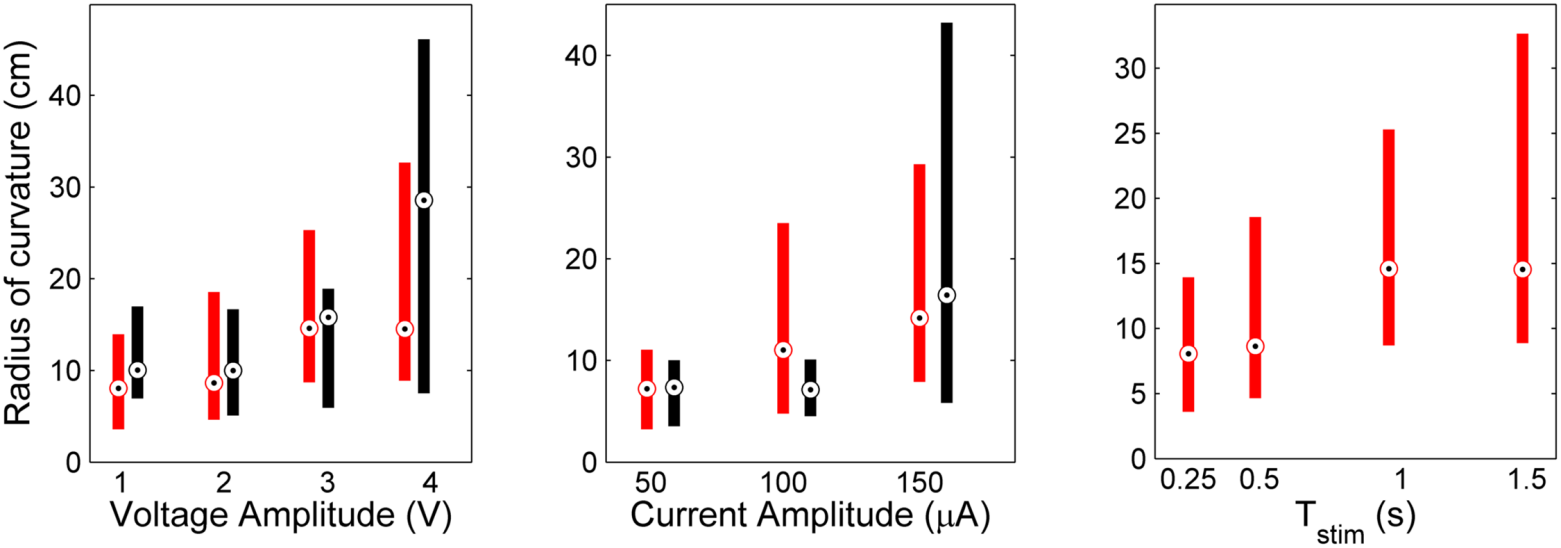
Radius of curvature increases with of stimulus amplitude, but not duration. Results are shown for bipolar VCS (left); CCS (center); and variable durations (right). Red and black color-code stimulus frequencies of 50 Hz and 200 Hz, respectively. Bulls eye markers indicate the median, and bars cover the 25–75th percentile. Variable amplitude tests had constant *T*_*stim*_ = 1.5 s, while variable duration tests were done with constant 3V, 50 Hz.

**Fig 9:**
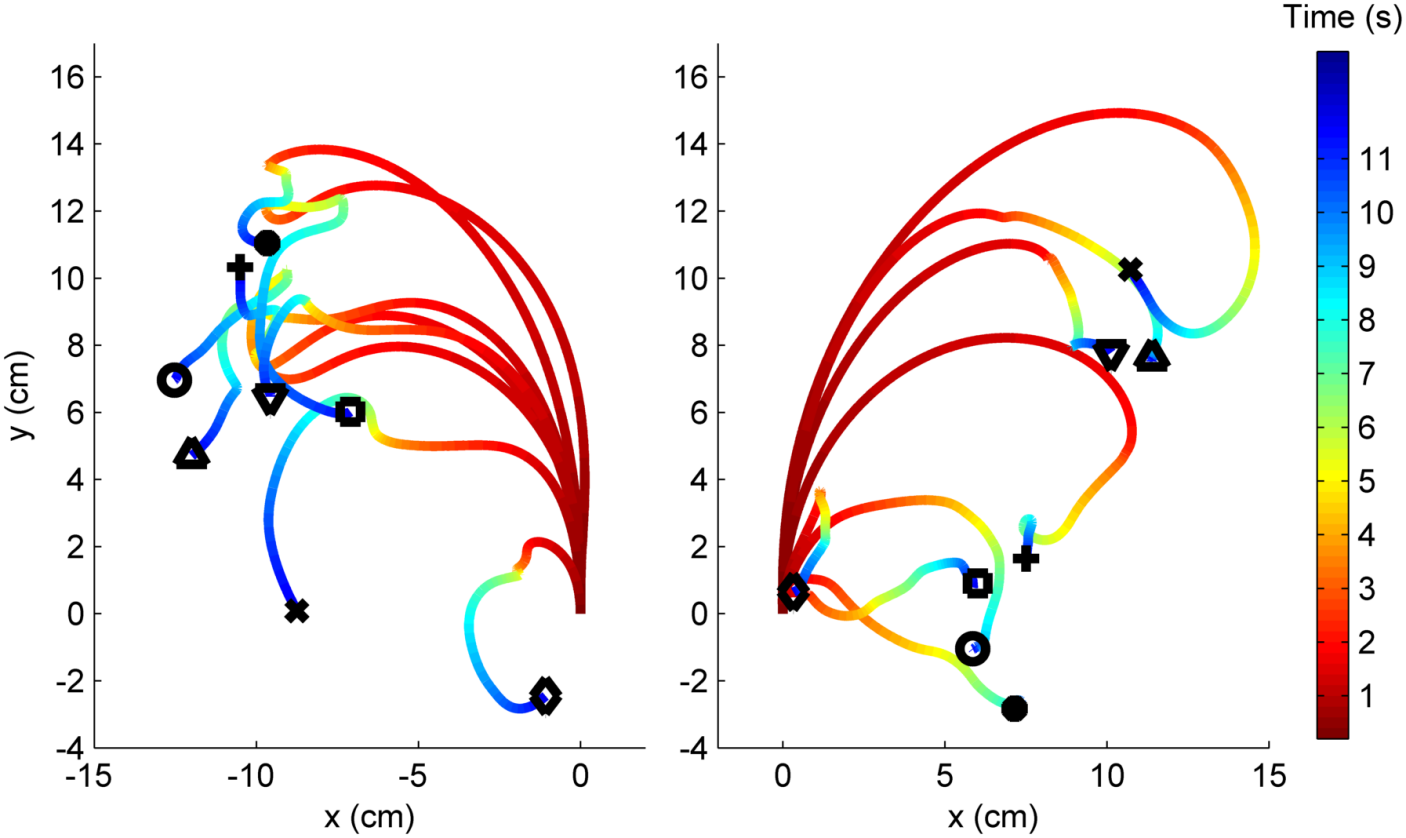
Trial-averaged fictive paths for a single test subject in response to 8 different stimulus parameter combinations. Path end point markers code for stimulus parameters: {∘, •, □, +} = 1–4V at 50 Hz; {⋄, ×, △, ▿} = 1–4V at 200 Hz. Stimuli are delivered to R antenna in left panel, and to the L antenna in right panel. The time scale is adjusted such that stimulus delivery occurs at *t* = 0 with all paths starting at the origin. Paths are color-coded for the the time at which a certain (*x*,*y*) coordinate was traversed.

In general, a statistically significant faster forward and slower turning motion resulted from increasing the amplitude of voltage and current pulses. Correspondingly, the fictive path radius of curvature (*R*_*c*_ = Δ*S*/Δ*θ*, [24]) was positively correlated to stimulus amplitude (Fig 8, Table 3). Fig 9 also illustrates this trend. Two exceptions were the turning responses Δ*θ* and Δ*ω*_*avg*_ for 200 Hz voltage pulse stimuli. The lack of overall correlation was attributed to the small angular motion in response to 1 V; an inverse relationship appeared to exist for 2–4 V amplitudes otherwise. The turning behavior of the MHC appears to be in contrast to *P. americana*, whose turning angle vs. stimulus amplitude was shown to increase in a linearly proportional manner when stimulating the cervical connective [25]. Lastly, it is worth noting that the that the median forward response was typically ≈3–5 body lengths (Δ*S* ≈15–25 cm) at a speed of ≈1 body length/s (Δ*v*_*avg*_ ≈4–5.5 cm/s).

For variable duration experiments, average velocity changes Δ*v*_*avg*_ and Δ*ω*_*avg*_ were found to be significantly positively correlated with stimulus duration. This result suggested a graded direct drive mechanism whereby the MHC quickly reaches and maintains a maximal velocity proportional to the stimulus strength for its duration, then decays on a relatively shorter time-scale when the stimulus turns off (e.g., see Fig 4, top row, 4th column). No statistically significant correlation between duration and either linear or angular displacement Δ*S* and Δ*θ* was observed. Therefore, the radius of curvature was not correlated with stimulus duration. That the path length traveled remained nearly constant across all durations tested suggested that that the MHC has a “pre-programmed” distance to travel once an escape is initiated. Lastly, it is worth noting that the largest turning angles occurred in response to the shortest duration stimuli (median Δ*θ* = 127.4 deg). Whereas shorter duration stimuli most often evoked the initial turn in the MHC, longer durations sometimes evoked a temporary seizing motion during stimulus delivery (legs splay and abdomen raises), followed by a run. One possible explanation is that, in some cases, longer duration electrical stimuli (*T*_*stim*_ ≥ 0.5 s) interfered with or overrode the natural neuromuscular activity patterns of the stereotyped turn-then-run escape response [9, 26].

While statistically significant correlations were commonly identified, *r* values tended to be somewhat low (0.1 < ∣*r*∣ < 0.4) due to the relatively large variances shown in Fig 7 (see also S2 Fig, S1 file). While S-R data were not necessarily linear, computing the regression slope values provides first-order information for building a quantitative S-R model, which may be useful for formulating more effective control strategies and minimizing the number of control inputs to navigate a MHC biobot through unknown terrain [27]. Given the relatively low *r* values, it must be recognized that this information is not precisely predictive of the locomotor response to a particular set of stimulus parameters on a single trial basis.

### Fictive paths

From a practical perspective, it is important to know not only how linear and angular velocity vary with stimulation parameters, but also how the fictive paths depend on them. For each stimulus parameter combination, fictive paths were typically highly stereotyped. Trial-averaged fictive paths (Eq 2) to various stimuli are illustrated in Fig 9. The path length traveled generally increased, but turns were not as tight for larger amplitude stimuli, in accord with the result that larger amplitude stimuli evoke faster walking but less turning, i.e., a larger radius of curvature. Contraversive turns were observed, as expected. Most of the motion occurred during the first ≈3–4 s following the stimulus onset; the locomotor response thus tapered off about 1.5–2.5 s after stimulus delivery had terminated.

**Fig 10:**
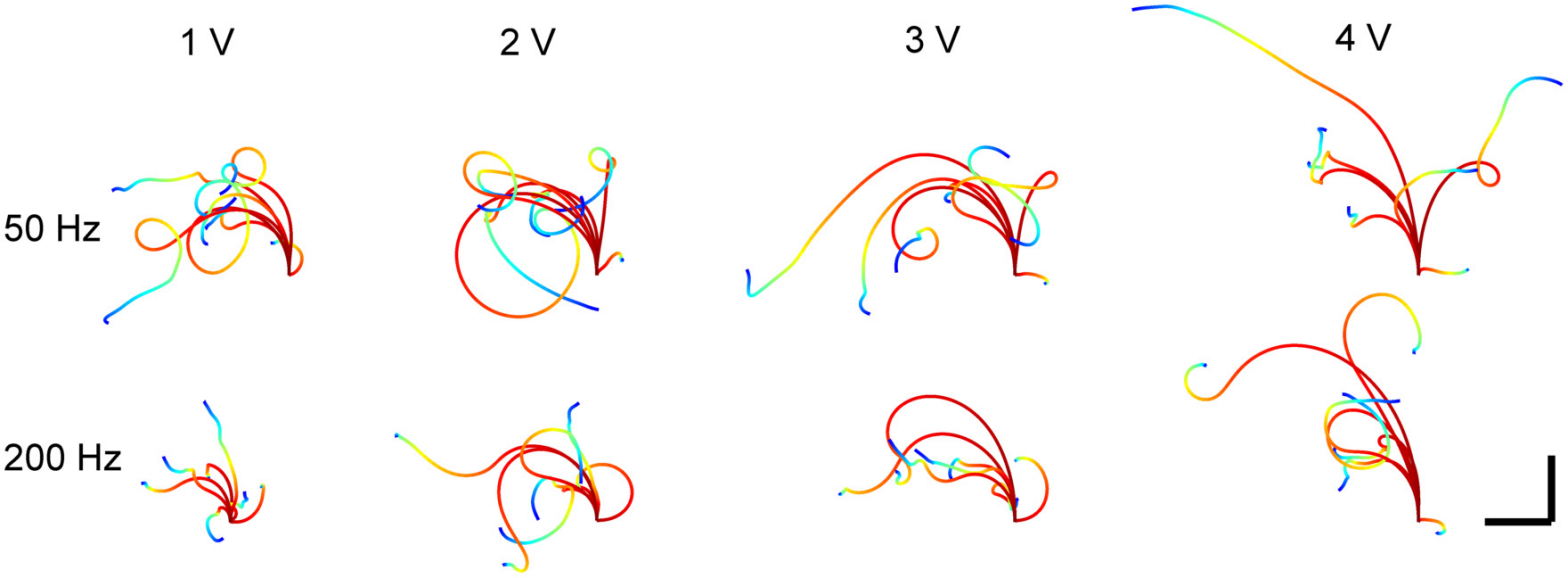
Trial-averaged fictive paths for 5 test subjects subject to various stimuli. Scale bar is 10 cm in both dimensions. Color-coding corresponds to the time at which a roach was at a certain position (see color bar in Fig 9).

To illustrate intersubject S-R variation, fictive paths for five insects are illustrated in Fig 10. For clarity, all paths shown are for stimuli delivered to the right antenna and both cerci. The expected contraversive turns to the left observed in the majority of cases.

### Power efficiency

**Fig 11:**
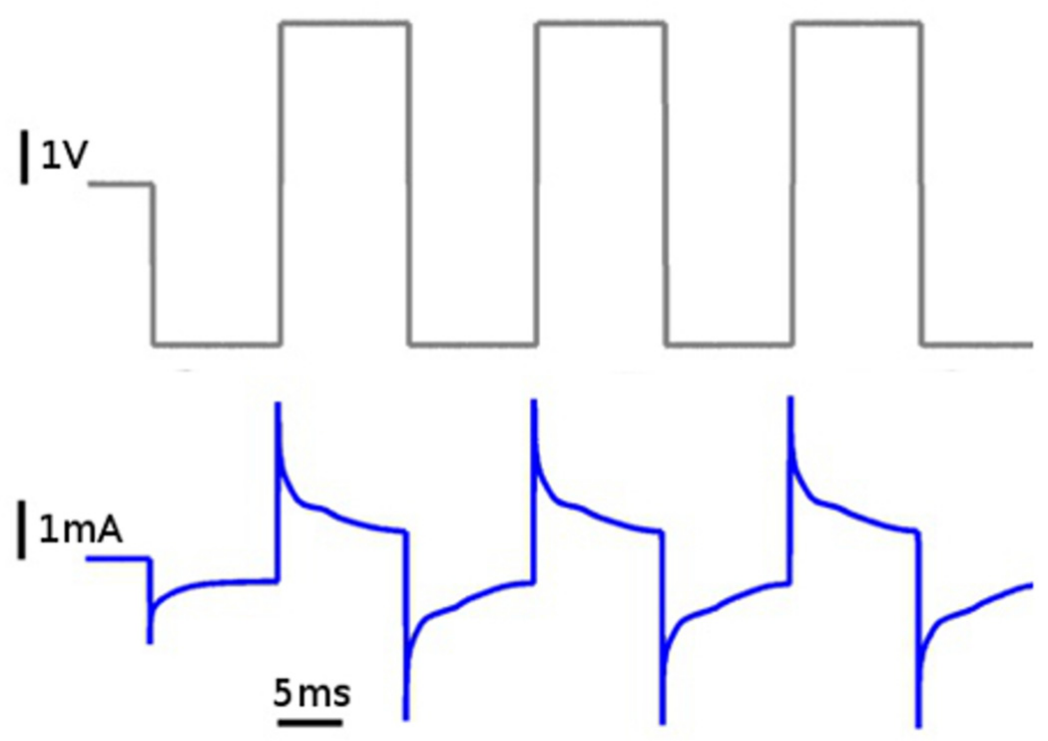
Example of simultaneous voltage (gray) and current (blue) measurement during bipolar voltage pulse stimulation. For the 3 V, 50 Hz waveform, the peak and time-averaged current amplitudes were 2.78 mA and 737 *μ*A, respectively.

For practical application with the MHC biobot, it is important to know how much power is dissipated during stimulation and how far an instrumented insect could travel on a single battery charge. An example of time-varying stimulus voltage and current is shown in Fig 11. Voltage pulses produce exponentially decaying current spikes, as expected, with an RC time constant of about 1 ms. For 3V, 50 Hz bipolar voltage pulses the time-averaged current and power were, respectively, 737±44 *μ*A and 2.2±0.13 mW (averaged over 60 trials).

Given a standard 2000 mAh LiPo battery and 0.5 s stimulus duration, and assuming both cerci plus one antenna are always stimulated, 6500 stimuli could be delivered before the battery is exhausted. Using an estimate of Δ*S* = 25 cm per stimulus (see Fig 7), a MHC biobot could potentially travel a distance of 1.62 km. In practical terms, a biobot could cover a seemingly useful amount of territory in a search and rescue operation, and this range could potentially be extended by implementing solar power for recharging batteries [13].

## Discussion

### Optimal electrical stimuli

This study identified neural-electrical stimulus parameter values that were shown to be effective for evoking a strong and consistent motor response in ≈50% of *G. portentosa* test subjects. Our results suggest that it may be possible to achieve a much higher success rate than the ≈10% previously reported by others [11, 12] in driving a MHC biobot along a desired path in a free-ranging arena, with electric stimuli to cerci and antenna delivered by a miniature wireless backpack mounted on the animal’s dorsal surface.

Importantly, our results showed that using bipolar stimuli avoided the significant problem of habituating locomotor responses, which has been commonly observed by us and other researchers when applying monopolar stimuli. Our findings suggest that habituation is unlikely to be attributed to neurotransmitters becoming depleted at synapses (e.g., [28]). Even so, it remains unclear how exactly bipolar VCS were superior in this regard. It could be the case that bipolar pulses avoid habituation by minimizing electrochemical damage at the tissue-electrode interface. However, monopolar 3V stimuli are not believed to cause electrochemical damage either [12].

Based on our findings, recommended optimal stimuli are charge-balanced VCS at 2 V and 50 Hz. This combination maximizes the success rate in evoking strong responses while minimizing habituation. Additionally, our results showed that graded tuning of fictive path trajectories could be achieved by varying the amplitude. One caveat is that primary turns may not occur in the expected (contraversive) direction for large amplitude (≥ 3 V) bipolar VCS.

To minimize power consumption, it is recommended to start with a 0.25 s duration, but increase as necessary, since *T*_*stim*_ ≥ 0.5 s was shown to be overall more effective. The neurobiological basis for increased effectiveness of longer duration stimuli may be explained as follows. It is known that in *G. portentosa* cercal wind puffs generate 40–50 spikes over an initial 250 ms interval, with a sustained response of 50–100 spikes/s in the connectives between the A2 and A3 abdominal ganglia [10]. Assuming that 1) a one-to-one correspondence exists between electric stimulus pulses and action potentials in ascending neural activity; 2) neural inputs can be integrated over longer time scales to generate a corresponding motor output; and 3) action potentials can be evoked in the cercal system by both positive and negative going current transients (i.e., by the corresponding rising or falling edges of voltage pulses), then the required duration of 50 Hz stimulus is ≥ 0.4 − 0.5 s. It should be noted that variable duration tests were done at 3 V and 50 Hz in this study, and the the interplay of duration with amplitude and frequency is not currently known. However, dependence on the latter seems unlikely, given that strong frequency dependence was not observed anywhere in this study.

### Comparison to previous work with MHC biobots

In contrast to a previous report in which no consistent effective single combination of stimulus parameters could be identified in amplitude and frequency ranges of 54–170 *μ*A and 40–105 Hz [11], we observed that 150 *μ*A current pulses at 50 Hz elicited reliable responses in 54% of test subjects. Statistically significant correlations were also identified for forward motion, turning angle, and radius of curvature vs. current pulse amplitude.

This study also quantified locomotion over a broader set of stimulus parameters in a relatively large number of test subjects, extending previous work that reported on turning behavior in response in monopolar 3.5 V voltage pulses with 0.2 s duration [4]. In order to make more direct comparisons of Δ*θ* (Table 4), results from our study for 1.5 s duration stimuli were time-scaled down to 0.2 s (e.g., × 0.2/1.5). Time scaling was justified on the basis that, in our study, the majority of motion occurred during stimulus delivery (e.g., see Fig 9). Bipolar VCS 2 V in amplitude were chosen for comparison because they are most closely equivalent to monopolar 3.5 V stimuli. Comparisons were made across the range of CCS amplitudes, as it was uncertain a priori which amplitude would most closely correspond to 3.5 V monopolar pulses. Scaled median turning angle results for VCS and CCS were in reasonable accord but slightly larger (+5.5 and +0.9 deg, respectively) than the value previously reported in [4]. It is also worth noting that, in *P. americana*, the mean initial turning angle in response to abrupt antennal contact is 97 deg [29], which falls within the range we measured for *G. portentosa* (Table 4).

**Table 4:** Comparison of turning angles to results reported in [4]. The mean turning angle and 25–75% range are given. For Stim 1, the range is estimated from data reported in [4].

Stimulus Parameters	Δ*θ* (deg)	Range (deg)
Stim 1: monopolar VCS, 3.5 V, 0.2 s	11.8	5.0–21.8
Stim 2: bipolar VCS, 2 V, 1.5 s	130	61.6–190
Stim 2 scaled to 0.2 s	17.3	8.2–25.3
Stim 3: bipolar CCS, (50, 100, 150) *μ*A, 1.5 s	95	79–111
Stim 3 scaled to 0.2 s	12.7	10.5–14.8

For turning rate, the median response to bipolar 2 V, 1.5 s stimuli was Δ*ω*_*avg*_ = 62.7 deg/s, larger than the 23 deg/s reported in [4]. This result may be explained by the observation that the MHC exhibits a monotonic angular acceleration throughout most (all) of the 1.5 s stimuli, thus a higher turning rate is achieved.

The radius of curvature of the initial turn was recently measured for *B. discoidalis* to be in the range of ≈3–10 cm [30], about one-half as large as *R*_*c*_ measured for *G. portentosa* in this study. The body length of *B. discoidalis* is about 1/2 to 2/3 as long as the MHC, thus the radius of curvature appears to scale with body length. Interestingly, whereas *R*_*c*_ increased with stimulus amplitude for the MHC, the opposite trend was observed in *B. discoidalis* [30]. This difference may be explained by the different stimulation strategies employed. In [30], the prothoracic ganglion controlling the front legs was directly stimulated. Evidently, different stimulation strategies may lead to qualitatively different outcomes.

### Comparison of stimulation strategies

In this study we employed neural-electrical stimulation delivered by microwires implanted in the antenna and cerci. An advantage of this method is that it is quick and easy to implement, and animal surgeries are minimally invasive. However, locomotion is driven by indirect means: the electrical stimulus must be processed by ascending neural pathways and ultimately converted into a sequence of impulses to motor neurons to coordinate an escape maneuver [9].

Various other strategies have been proposed to control biobot motion more directly. Giampalmo et al. showed that grasshoppers could be made to jump by stimulating the T3 metathoracic ganglion which controls the large hind legs [7]. More recently, Sanchez et al. demonstrated that a graded walking and turning response in *B. discoidalis* cockroaches could be generated via direct stimulation of the prothoracic ganglion, which controls movements of the first pair of legs [30]. However, one potential drawback is that proper placement and implantation of electrodes into the ganglia is somewhat more difficult. Nonetheless, an admirable ≈60% success rate was achieved, which is similar to the success rate achieved in the present study with optimal bipolar stimuli.

Direct muscle stimulation is another strategy previously implemented for both terrestrial and flying motion in beetles [3, 5]. Sato et al. recently demonstrated fine and graded control of turning during free-flight in beetles, but is difficult to achieve, in general, without specific knowledge about the precise coordination of small muscle groups [31]. Cao et al. have demonstrated closed-loop control over an insect leg by stimulating three antagonistic muscle groups [5]. A potential drawback of this method is the hardware and algorithms required to coordinate stimulating up to 18 separate muscle groups (3 per leg) to generate walking and turning in an insect.

Lastly, Visvanathan et al. developed a piezoelectric heating element for tactile stimulation, which successfully elicited motor responses in a range of insects, including the MHC [32]. However, this method was power intensive, consuming 330 mW per stimulus. By contrast, neural-electric stimuli were shown in this study to be on the order of 100× more power efficient.

### Limitations and future work

There were several limitations with this study that should be made clear. For some experiments, conclusions were drawn from experiments with as few as 12 test subjects. Nonetheless, the pool was sufficiently large to observe sensible trends in S-R relationship. Significant intersubject variability was noted throughout these experiments. No attempt was made to control for the hydration and nutrition state of the roach, or the phase of day/night cycle. In practical terms of deploying a swarm of MHC biobots, this variability may make it difficult to predict precisely what a particular biobot’s actual response will be. Scaling locomotion metrics to length, mass, or the product thereof, did not account for the observed variability. No systematic differences have been noted comparing male vs. female responses.

Another possibility we considered was the test subject’s internal temperature. It is well known that the MHC’s level of physical activity increases with temperature [33]. While we ascertained test subjects reached a temperature of about 35–37 C (measured on exoskeleton with infrared thermometer) prior to the start of an experiment, we made no attempt to control the temperature over time other than crudely placing a warming light above the motion tracking system apparatus. We also noted that it became much more difficult to evoke a motor response when the external temperature about 30 C. One future consideration for deploying biobots in a colder environment (e.g., earthquake rubble) is that they may require a “heating blanket” to remain active.

Whether and how other sensory inputs influenced variability in motor response merits further consideration. During our experiments, we made no attempt to precisely control ambient light levels. Such visual inputs converge on the thoracic interneuron circuitry that generates motor neurons output in *P. americana* [34]. Analogous neural circuitry is likely wired in the MHC too. The MHC is known to communicate through hissing at audible frequencies in socially context dependent manner [35]. Whether and how auditory input— specifically, the audible hiss of the pressurized air floating the trackball—could be integrated into the MHC locomotor response observed in this study remains unknown.

Another candidate to explain variability was the exact placement of an electrode relative to sensory neurons that transduce the electrical stimulus into a neural signal and ultimately into a motor response. While we made every effort to implant electrodes in the same location in across all test subjects, inevitably there was some variation in placement on a millimeter size scale. Therefore, it is not unreasonable to think that different neurons were stimulated in different test subjects, which could account, in part, for some variability. A careful study of this issue has yet to be undertaken. Even so, it may be impractical to expect electrode implantation to become so precise as to target particular neurons.

## Conclusion

In summary, this work identified a set of effective electrical stimulus parameters for evoking stereotyped locomotion in the MHC. Stimulation of the cerci and antenna is an easy-to-implement and power-efficient method to generate a graded motor response. While this method has been historically problematic, primarily due to habituation, the present study has elucidated that bipolar voltage stimuli are effective for generating sustained and consistent responses with a success rate of ≈50%. We note that the constant frequency stimuli used in this study do not capture the time-varying spike rate of the natural cercal system neural response to wind puffs [10]. A future study will investigate whether there is a benefit to using more complex stimuli with time-varying frequency.

This study also quantified the MHC locomotor response to various neural-electric stimuli. To the best of our knowledge, this is currently the most complete description of its kind. It has previously been remarked that such detailed knowledge of the stimulus-response relationship could lead to optimized stimulation strategies for biobot applications [4]. Therefore, the present results may promote more fully realizing the potential for biobot applications.

## Supporting Information

S1 File This supporting information describes the automated method for clustering multiple phases of turning response, and provides more details on S-R model intersubject variation. (PDF)

S1 Fig Example of clustering turns with modified FSMEM. Primary responses are colored blue, secondary are red according to the time the response was the initiated and the turning angle.
(TIF)

S2 Fig Intrasubject variation in responses to bipolar voltage and current pulses. The standard deviation (S.D.) is plotted in a format analogous to Fig 7.
(TIF)
