# Supplementary material for: Effective Stimulus Parameters for Directed Locomotion in Madagascar Hissing Cockroach Biobot
Source: PLoS One. 2015 Aug 26;10(8):e0134348. doi: 10.1371/journal.pone.0134348 (PMC4550421; doi:10.1371/journal.pone.0134348)
Supplement: S1 File — (PDF) [file pone.0134348.s001.pdf]

Effective stimulus parameters for directed locomotion in Madagascar hissing cockroach biobot

Jonathan C. Erickson[1], María Herrera[1¶], Mauricio Bustamante[1¶], Aristide Shingiro[1&], Thomas Bowen[1&, #a]

1 Department of Physics-Engineering, Washington and Lee University, Lexington, Virginia, United States of America

[¶] These authors contributed equally to this work.

[&] These authors also contributed equally to this work.

[#a] Current Address: Plant Biomechanics Group, University of Freiburg, Freiburg, Germany

*** E-mail: ericksonj@wlu.edu (JCE)**

Supporting Information

Automated clustering of multiple phases of turning response

Figure 1 illustrates the motivation for using the FSMEM automated clustering method to identify multiple phases of a turning response. The timing and duration of separate turning phases was variable in response to repeated stimuli with the same combination of parameter values. Therefore, the time at which a response was initiated alone was often found to be insufficient to properly identify primary responses.

Therefore, we clustered primary turns based on a cloud of data points specifying when a turn was initiated as well as the magnitude and direction and of the turn: $(T_{turn,j},\, \Delta\theta_j)$, where where $j = 1,\ 2,\ \ldots$ indexes the turning phase.

The free split merge expectation maximization (FSMEM) algorithm [1] was applied to this cloud of $(T_{turn,j},\, \Delta\theta_j)$ data points to cluster turns associated with primary and secondary responses (1). It is important to note that we used the digitally low-pass filtered data for $\omega(t)$ to determine the time at which a turn was initiated. Given the smoothing inherent with filtering, some response times were marked as occurring before the actual onset of stimulus delivery ($t = 3000$ ms).

Details of the FSMEM algorithm can be found in [1]. Briefly, the algorithm assumes a mixture of Gaussians can appropriately model the cloud of 2-D data points, and the number of clusters dynamically updates.

One important modification we made to increase the chances that proper clustering occurred was to initialize the process with a judicious selection of means and standard deviations. This was done by implementing the minimum error thresholding [2] on a histogram of the times at which turning phases were initiated (”Time response initiated” in Figure 1). This method yields an initial threshold value partitioning the cloud of data points into 2 clusters. The centroid and standard deviation in along both dimension (turn angle and response initiation time) was computed for each subset of points and passed into the FSMEM algorithm. In practice, the initial threshold selection made a good first approximation clusters differentiating the primary and secondary (and higher order) turning phases. This fully automated modified FSMEM method succeeding in properly identifying primary turns in the majority of cases. In some rare case, we had to use a semi-automated method to correct FSMEM clustering. In summary, FSMEM was highly beneficial for rapid and automated identification of turning phases.

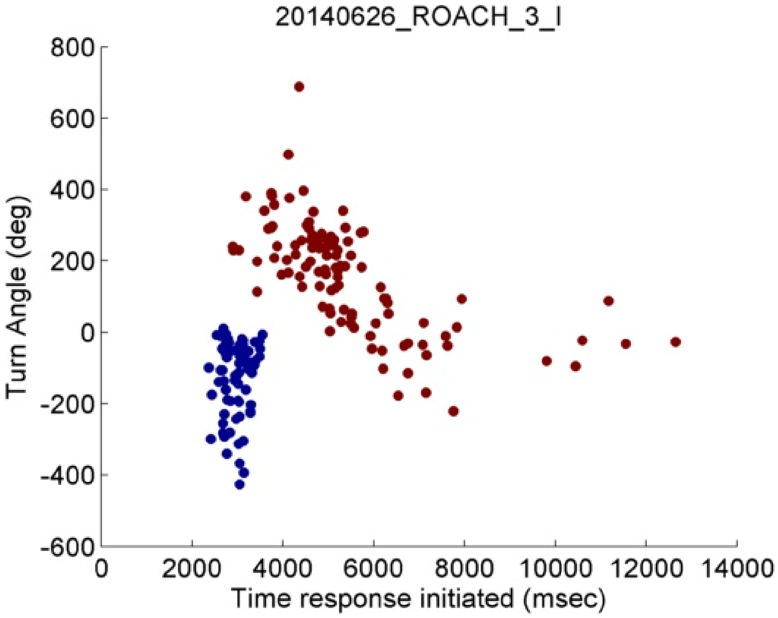

Figure 1. Example of clustering turns with FSMEM. Primary responses are colored blue, secondary are red.

Locomotor metrics: sources of variation

Given the significant variation observed pooling results across all test subjects, it is natural to quantify the intrasubject variation: How much jitter is observed in the response to repeated presentations of the same stimulus for a single test subject?

For each test subject, we computed the standard deviation (S.D.) to repeated stimuli with constant parameters (Fig. 2). Although the distribution of response metrics may not strictly follow a normal distribution, the S.D. can still provide a reasonable estimate for the width of the S-R distribution for a single test subject. For example, for bipolar 2V, 50 Hz voltage pulses, the turning rate $\Delta\omega_{avg}$ is distributed over a (median) width of ± 27.1 deg/s, while the $\Delta\theta$ is distributed over a (median) of about ± 80 deg. In large part, intrasubject variation accounted for the group variation shown in the main manuscript, Fig. 7.

Overall, no set of stimulus parameters offers clearly better precision in responses to repeated stimuli. It was also apparent that some test subjects exhibited very regular responses (less jitter), while others were more highly variable. Subjects with more predictable responses (less jitter) are desirable, but it is not clear to identify such subjects a priori.

References

1. Wagenaar DA, Pine J, Potter SM (2004) Effective parameters for stimulation of dissociated cultures using multi-electrode arrays. Journal of neuroscience methods 138: 27–37.

2. Kittler J, Illingworth J (1986) Minimum error thresholding. Pattern recognition 19: 41–47.

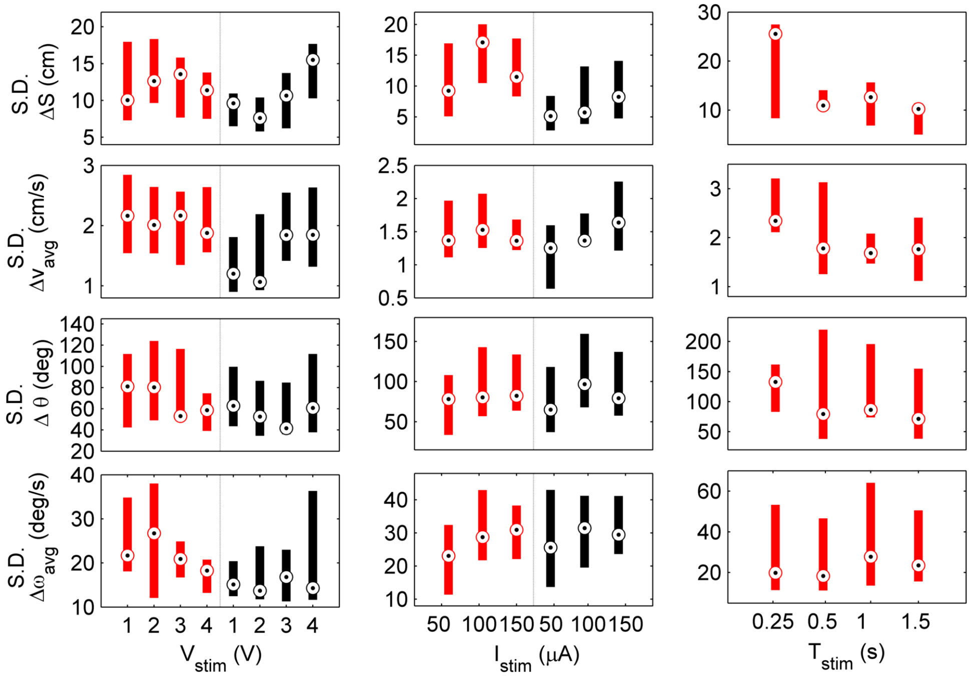

Figure 2. Intrasubject variation in responses to bipolar voltage and current pulses. The standard deviation (S.D.) is plotted in a format analogous to Fig. 7.
