# Supplementary figures and images for: Effective Stimulus Parameters for Directed Locomotion in Madagascar Hissing Cockroach Biobot
Source: PLoS One. 2015 Aug 26;10(8):e0134348. doi: 10.1371/journal.pone.0134348 (PMC4550421; doi:10.1371/journal.pone.0134348)

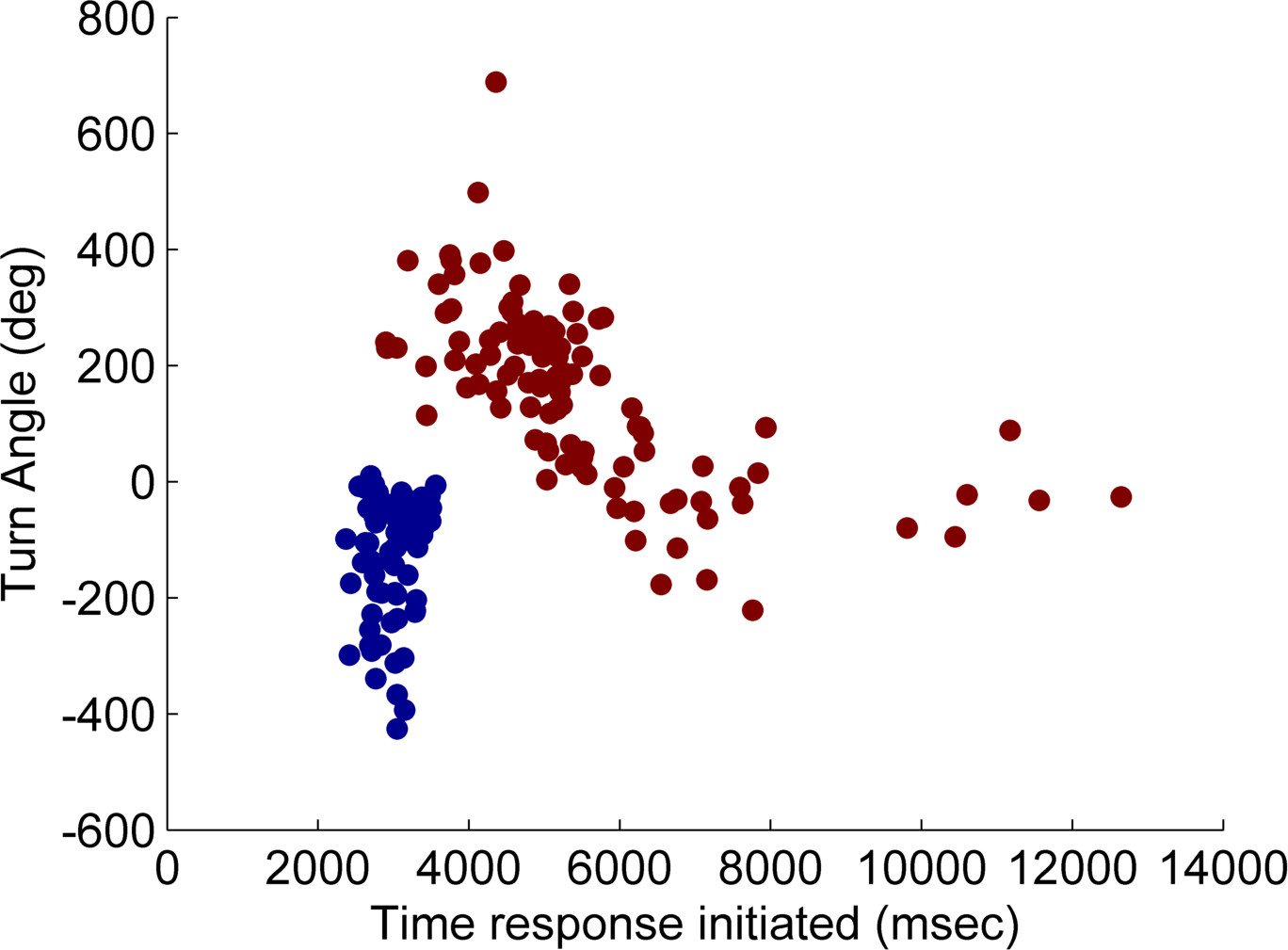

Supplement: S1 Fig — Primary responses are colored blue, secondary are red according to the time the response was the initiated and the turning angle. (TIF) [file pone.0134348.s002.tif]

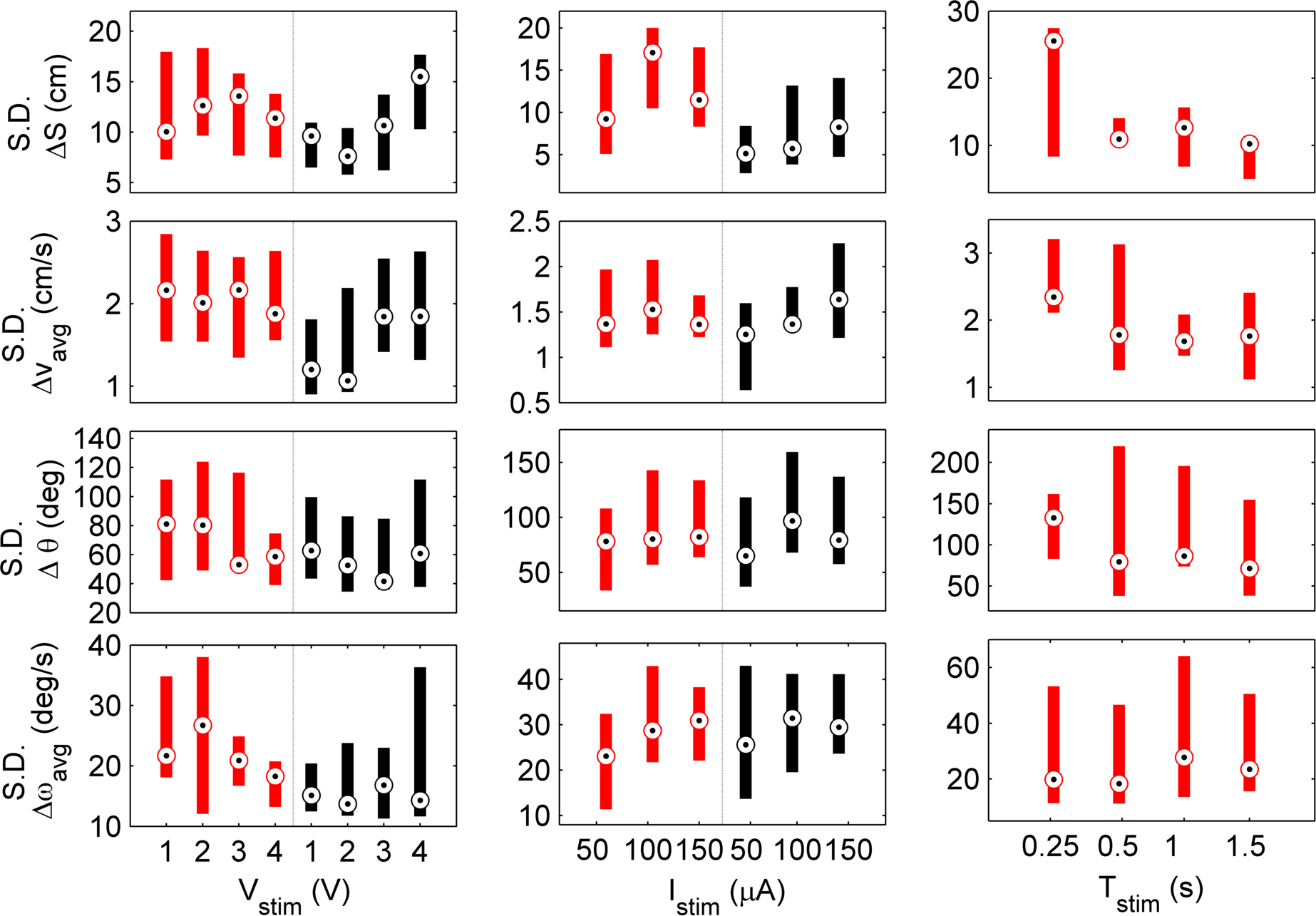

Supplement: S2 Fig — The standard deviation (S.D.) is plotted in a format analogous to Fig 7. (TIF) [file pone.0134348.s003.tif]
